# Investigating the Relationship between Self-Injurious Behavior, Social Deficits, and Cooccurring Behaviors in Children and Adolescents with Autism Spectrum Disorder

**DOI:** 10.1155/2012/156481

**Published:** 2012-11-07

**Authors:** Paul Waters, Olive Healy

**Affiliations:** School of Psychology, National University of Ireland, Galway, Ireland

## Abstract

Research suggests that self-injurious behavior (SIB) is related to social deficits and cooccurring problem behaviors in children and adolescents with autism spectrum disorder. A sample of 95 participants with ASD was assessed on presence and frequency of SIB (Behavior Problems Inventory), social deficits (the Matson Evaluation of Social Skills with Youngsters-II) and cooccurring problem behaviors (ASD-Comorbidity-Child version). A model was created and tested to explain the relationship between these variables. Results showed that the model was acceptable in presenting the relationships between these variables. This information could be used to help predict which individuals are at risk of developing further cooccurring behavioral problems and determine risk markers for the development of social deficits.

## 1. Introduction 

Self-injurious behavior (SIB) is possibly the most acute of all the behavioral problems that are commonly present in individuals with neurodevelopmental disorders [1]. SIB is often described as a chronic form of challenging behavior, the aetiology of which has been poorly understood [2, 3]. It is a challenging behavior for parents, professionals, and direct care staff [4]. Jones [5] divided SIB into two classes on the basis of frequency: “stereotyped self-injurious behavior” (SSIB) referred to self-injury that is displayed in a repetitive, invariant way and which is typically exhibited at a high rate, and “self-aggression” which is described as having a stronger link with environmental contingencies and is often exhibited at a lower rate. The topography of SIB varies from case to case, head hitting against objects or hands and hand biting, skin scratching, and hitting other parts of the body being the most common [6, 7]. 

 Autism spectrum disorder is a prevalent developmental disorder which has grown in prevalence over the past decade [8]. Research has shown that abnormal behaviors are common in children with ASD [9]. Murphy et al. [10] found that 48% of children with ASD exhibited a form of SIB, with the most common topography being hand biting. SIB was also seen to cooccur, in the majority of cases, with another form of challenging behavior such as aggression or stereotypy. Baghdadli et al. [11] found that 50% of 222 children with ASD under the age of 7 years engaged in SIB, higher than those with just ID. 

Increased prevalence rates of SIB have been shown to be associated with a range of personal and environmental factors such as age, restrictiveness of environment, and impairments of mobility and communication [6]. Individuals diagnosed with ASD are at a higher risk of developing SIB than those without the disorder [9]. Baghdadli et al. [11] reported that increased severity of ASD resulted in greater levels of SIB. Challenging behaviors, such as self-injury, may persist throughout adulthood, especially for those diagnosed with ASD [12]. Furthermore, those persons with comorbid problems of ASD and ID are at increased risk for the presence of SIB [13]. 

 SIB has often been treated as a discrete behavior independent from other forms of challenging behavior. However, Matson and Wilkins [14] reported a significant relationship between SIB and physical aggression, property destruction, stereotypy, and sexually inappropriate behavior. It has also been suggested that SIB is a complex behavioral problem involving cooccurring behaviors [15]. A link between SIB and other maladaptive behaviors has also been reported. Nøttestad and Linaker [16] showed that SIB is a significant predictor of aggressive challenging behavior, and Matson et al. [4] found that irritability may be predictive of SIB in individuals with severe and profound intellectual disabilities.

 Challenging behavior, including SIB, is often associated with negative personal and social consequences [10]. Because persons with ASD often present with additional maladaptive behaviors such as physical aggression [17], this cooccurrence of challenges may further impact on negative social interaction. The number of atypical behaviors shown in ASD is strongly related to the severity of the social deficits and repetitive behaviors present [9, 18]. 

Furthermore, Matson and Rivet [12] found that individuals with ASD and cooccurring psychopathology had more impairment in communication and social skills than those with ASD alone. It has been shown that the presence of challenging behaviors in individuals with developmental disorders is a predisposing factor to social impairments [6, 19]. Social deficiencies have been found to be pervasive in persons with ASD, and the presence of challenging behaviors can further impede daily functioning [20]. 

Social skills are a significant component of an individual's behavior and are necessary for effective interpersonal communication [20]. Social skills deficits are commonly associated with a variety of disorders including ASD [21] and ID [19]. Positive social behaviors have been linked to improved developmental outcomes for the individual, and social deficits have been shown to impact on the development of further difficulties [22]. 

 In this paper, a model showing the interaction between SIB, cooccurring behaviors, and their effect on the social repertoires of children and adolescents with ASD is proposed. SIB has been shown to be related to cooccurring maladaptive behaviors. These behaviors, along with SIB, can negatively affect an individual's social skills. The model was developed to show the effect of this negative impact on social behaviors using a number of standardized measures. Other unobserved variables that could also impact on the previous variables were taken into account. The goal of this paper was to evaluate the effects of SIB and cooccurring challenging behavior on social skill repertoires within a structural equation model as the analytical technique.

## 2. Method

### 2.1. Participants

A total of 106 children and adolescents with ASD completed assessments for this study. They were selected from several different service providers including special schools, ASD units attached to primary schools, and home-based intervention programs. As part of the inclusion criterion, all participants were scored on the self-injurious behavior subscale of the Behavior Problems Inventory (BPI-01). Participants were screened and accepted onto the study if they scored more than zero in at least one item on the BPI-01 SIB subscale, indicating the presence of SIB. Of the 106 respondents, 95 were included. The mean age of the participants was 10.8 years (minimum age 3 years; maximum age 16 years). The frequencies of Intellectual Disability (ID) levels are as follows: profound = 1, severe = 6, moderate = 27, mild = 18, average = 7, and unknown = 36. Two participants had a hearing impairment, one had a visual impairment, and 12 were epileptic. The mean amount of time the participants had been engaged in services was 44 months (SD = 37). All participants were diagnosed with ASD, based on DSM-IV criteria, by psychologists independent of their service provider. Staff members and/or caregivers acted as informants and completed the measures. 

### 2.2. Measures

#### 2.2.1. The Behavior Problems Inventory

The Behavior Problems Inventory (BPI-01; [23]) is a respondent-based behavior rating assessment for self-injurious, stereotypic, and aggressive/destructive behavior in intellectual disabilities and other developmental disabilities. It is a 52-item scale, with items rated on frequency and severity. The BPI-01 was found to be a reliable (retest reliability, internal consistency, and between-interviewer agreement) and valid (factor and criterion validity) behavior rating instrument for problem behaviors in intellectual and developmental disabilities with a variety of potentially useful applications [23]. Participants were assessed on prevalence, topography and severity of SIB using the BPI-01 SIB subscale, consisting of 15 items describing topography and records frequency and severity. The presence of the problem behavior must be present for two months, prior to administration of the instrument, in order for it to be scored. Each item is scored on a five-point frequency scale (0 = never, 1 = monthly, 2 = weekly, 3 = daily, and 4 = hourly) and a four-point severity scale (0 = no problem, 1 = a slight problem, 2 = a moderate problem, and 3 = a severe problem).

#### 2.2.2. ASD-Comorbidity-Child Version

The cooccurrence of additional behaviors such as tantrums, depressed, and repetitive behavior was assessed using the ASD-Comorbidity-Child version (ASD-C-C; [24]). It is a 39-item, informant based assessment; the caregiver rates each item in severity. The scale comprises of (0 = not a problem or impairment, 1 = mild problems or impairment, and 2 = severe problems or impairments). Scores are added together for a total score. Scores are divided across seven behavior subscales; tantrum behavior, repetitive behavior, worry/depressed, avoidant behavior, undereating, conduct, and overeating.

#### 2.2.3. Matson Evaluation of Social Skills with Youngsters-II

The Matson Evaluation of Social Skills with Youngsters-II (MESSY-II; [25]) is a social skills measure for 2–16-year olds. It is a 64-item assessment using a Likert scale, 1 (not at all)–5 (very much). The assessment yields three factors; hostile, adaptive/appropriate, and inappropriate assertiveness/overconfident scales. Factor scores are obtained by summing the Likert rating for each item in the scale. A high score on the hostile and/or inappropriate assertiveness/overconfident factors indicate the presence of poor social skills, while a low score on the adaptive/appropriate factor indicates a lack of positive social skills. The MESSY-II reports a high degree of internal consistency with alpha coefficients of  .84 for the 2- to 5-year olds,  .93 for the 6- to 9-year olds, and  .93 for the 10- to 16-year olds [26]. It is completed by a caregiver and provides information on communication and general social skills. 

### 2.3. Procedure

 Frontline staff within the service providers acted as informants and were given specific instructions on the nature of the study and how to complete the measures with regard to the service users in their care. Informants were staff members who worked in the service for a minimum of 6months and were designated key workers with participants. They completed the rating scales with guidance from the researchers. All completed assessments were then returned to the researchers and scored. 

### 2.4. Data Analysis

The data were analysed using SPSS version 20 and Analysis of Moment Structures (AMOS) version 20. A structural equation modelling (SEM), a type of multivariate analysis, was applied to confirm the theoretically built model containing the domains of frequency of SIB, severity of SIB, hostile behaviours, adaptive/appropriate behaviours, inappropriately assertive behaviours, and co-morbid behaviours. The model was designed with a defined research question. Frequency of SIB, severity of SIB, and cooccurring behaviours were entered as exogenous variables. Social skill was entered as a latent variable. Hostile, adaptive/appropriate behaviour and inappropriately assertive behaviour were entered as endogenous variables. Correlations between error terms were entered, and constraints were put on parameters to the model. Then, measures of model fitness and modification indices of AMOS were obtained. The chi-square statistic provides a test of the null hypothesis that the theoretical model fits the data. The following acceptable threshold levels were taken from Hooper et al [27]. The suggested *P* value for this test should be more than 0.05. The criteria for model fit used were relative chi-square statistic of less than or equal to 2.0, Goodness of Fit Index (GFI) statistic of equal to or greater value than 0.95, Comparative Fit Index (CFI) of equal to or greater value than 0.95, and Root Mean Square Error of Approximation (RMSEA) less than or equal to 0.7. The estimates and significant levels of correlation and regression parameters from the fit model were then presented. 

## 3. Results

### 3.1. Characteristics of Observed Variables

The mean (SD) age of the participants was 10.8 (4) years. The mean (SD) score of the frequency of self-injurious behaviour was 7.64 (5.89) and for severity of self-injurious behaviour was 5.76 (5.88). Mean scores of the MESSY-II subscales were as follows: hostile = 36.27 (SD 11.95); adaptive/appropriate = 28.1 (SD 10.21); inappropriate assertiveness/overconfident = 20.57 (SD 6.71). The mean (SD) score of the sum of all ASD-C-C subscales was 32.64 (15.4).

### 3.2. Correlation Coefficients

Table 1 presents the correlation matrix of the manifest variables included in the structural equation model. Frequency of SIB was positively correlated with severity of SIB (*r* = .565, *P* < .01), negatively correlated with the MESSY-II adaptive/appropriate subscale (*r* = −.206, *P* < .05), positively correlated with the MESSY-II inappropriately assertive subscale (*r* = .212, *P* < .05), and positively correlated with the sum of all ASD-C-C subscales (*r* = .257, *P* < .05). Severity of SIB was positively correlated with the sum of all ASD-C-C subscales (*r* = .324, *P* < .01). The MESSY-II hostile subscale was positively correlated with the MESSY-II adaptive/appropriate subscale (*r* = .481, *P* < .01) and positively correlated with the MESSY-II inappropriately assertive subscale (*r* = .728, *P* < .01). The MESSY-II adaptive/appropriate subscale is positively correlated with the MESSY-II inappropriately assertive subscale (*r* = .421, *P* < .01).

### 3.3. Structural Equation Model

**Figure 1:**
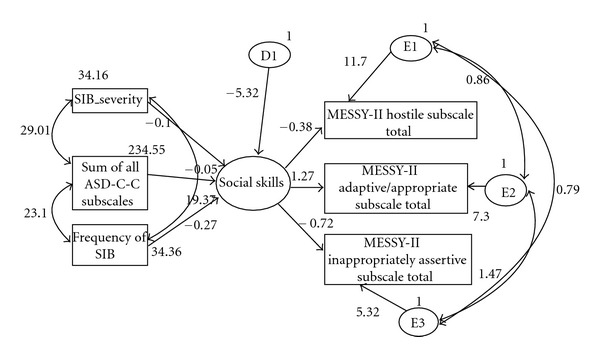
Structual equation model for SIB, cooccuring behaviours and social deficits for children and adolescents with ASD. *N* = 95*. χ* ^2^ = 3.415, df = 4, *P* = .491, relative chi-square (.854), GFI (.988), CFI (1.000), RMSEA (.000) and *P* Ratio (.267). All pathways were significant with *P* < .05, and standardised coefficients were shown next to each pathway. Latent variables in oval and manifest variables in rectangle boxes.

Figure 1 shows significant pathways of the final model and their goodness-of-fit indices. A single latent variable, social skills, was included in the model. Three exogenous variables were used for this latent variable. These included frequency of SIB, severity of SIB and total sum of ASD-C-C scores. These manifest variables acted as predictors for social skills, which in turn acted as a predictor for the endogenous variables, the MESSY-II subscales (hostile, adaptive/appropriate, and inappropriately assertive).  Table 2 shows the estimates of the final model. Small, negative associations were shown between the latent variable social skill and its exogenous variables, with low values of standardized coefficients ranging from −0.008 to −0.274. Strong-to-moderate positive and negative associations were shown between the latent variable and the endogenous variables, ranging from −0.381 to 1.266. Error terms were entered for each of the endogenous variables, and a disturbance was included for the latent variable. The error terms had strong associations ranging from 5.318 to 11.7, while the disturbance showed a standard coefficient of −5.232. The diagnostics of the model indicated that all error terms for the MESSY-II subscales (hostile, adaptive/appropriate, and inappropriately assertive) were intercorrelated. The measures of model fitness were as follows: chi-square for the model (*χ* ^2^ = 3.415, df = 4, *P* = .491), relative chi-square (.854), GFI (.988), CFI (1.000), RMSEA (.000), and *P* Ratio (.267). All indices suggest that the presented final model reasonably fits the data.

## 4. Discussion

This study aimed to confirm a model that displays the interaction between SIB, cooccurring behaviours, and social deficits in children and adolescents with ASD. The results of this study present a model that displays the interaction of SIB and cooccurring behaviours on social skill repertoires. The average age of participants in this study was 10.8 years and all attended a school that catered specifically for individuals with ASD. All participants presented with SIB as a problem behavior. Participants scored on average in the lower end of the scale regarding hostile and inappropriately assertive behaviour, but scored in the severe impairment range for adaptive/appropriate social skills.

A deficit in social functioning is a core feature of autism, and there exists a myriad of literature documenting the various effects of such deficits on children diagnosed with this developmental disorder. The fundamental diagnostic characteristics of the disorder vary across a spectrum, and social skills deficits can be diverse. Social skills are an important repertoire necessary for effective interpersonal communication [20]. The severity of social impairment in autism can be impacted by a number of variables including the presence of maladaptive or challenging behavior. Autism is often accompanied by complicated cooccurring conditions that are symptomatic of various forms of challenging behaviour including disruptive behaviour, property destruction, and aggression, and self-aggression. Research has shown that this subgroup of the population may present with high rates of behavioral and emotional problems, including mood swings, aggression, tantrums and self-aggression [28, 29]. The presence of cooccurring problems including challenging behaviour increases the complexity of the symptoms that present at diagnosis and across development. Although not being a diagnostic factor, the presence of challenging behaviors by persons with autism is prevalent with many evincing at least one form of challenging behavior [10, 30, 31].

The complex relationship between cooccurring problem behavior in autism and social skill impairment can be affected by moderating variables such as frequency and severity of self-injury. The current study sought to elaborate the underlying structure of interrelationships among predictors and outcome variables. Social skills were entered as a latent, or unobserved, variable. Social skills were predicted by the exogenous variables frequency of SIB, severity of SIB, and cooccurring behaviour. Social skills in turn predicted social deficits. This model shows that the frequency of SIB, severity of SIB, and cooccurring behaviour measured by the ASD-C-C all have a negative impact on social skills. The relationship between these behaviours and social skills deficits has been noted in previous research [6, 10, 12, 19]. A possible explanation is that challenging behavior becomes a barrier for engaging in meaningful social interaction due to its aversive nature [4, 7, 12]. The model also showed that there was a significant amount of covariance between the severity of SIB and cooccurring behaviours and between the frequency of SIB and cooccurring behaviours. Once again, this relationship has been noted in previous research [4, 16]. 

The symptoms of autism can affect an individual across his development and lifespan. The presence of cooccurring problems, such as conduct or behavior disorders, can greatly impact the complexity of the core symptoms of the condition. It has been demonstrated that behavioral problems in autism are prevalent with a range from 35.8% to 94.3% [32] and a large number of studies identifying at least half of participants with autism engaging in one or more challenging behaviors (e.g., [10, 33]). Self-injury is often observed as one form of challenging behavior in autism and can threaten both personal safety and the safety of others and can also significantly impact on one's exposure to greater numbers of learning opportunities including social exchanges. Indeed, the negative impact of both the presence of self-injury and cooccurring problem behaviors on social deficits demonstrated in the present sample indicates a need to address such problems in order to increase appropriate social abilities in this population.

Additionally, the presence of self-aggression can impact significantly on a person's quality of life because such behaviour often results in the exclusion of the person from many activities and community excursions. This can result in limitations to greater learning opportunities and new experiences within varied and enriched environments. Indeed, the long-term effects of severe self-injurious behaviour in autism can include greater risk for institutionalization, more intrusive interventions involving forms of physical restraint, prescription of antipsychotic medication, and limited opportunities for educational, social, and vocational programming (see, e.g., [34]). These effects can further increase the risk for limited social ability across the lifetime. 

 Within the structural equation model, hostile, adaptive/appropriate, and inappropriately assertive behaviours were entered as endogenous variables. The model showed that these variables were significantly affected by the social skill variable. When social skill increases, both hostile and inappropriately assertive behaviours decrease. When social skill decreases, inappropriately assertive behaviours increase. The direction of these relationships illustrates the importance of social abilities in regulating appropriate and inappropriate social behaviours.

In recent years there has been an increased emphasis on providing children and adolescents with ASD with more opportunities for inclusion and socialization through access to regular classroom environments. Unfortunately, many children with ASD do not readily or spontaneously demonstrate appropriate social interaction with their peers [35], rather, these children often require very in-depth specialized intervention to promote positive and meaningful integration within such classrooms. There is growing empirical evidence to support the effectiveness of behavioral interventions to teach, maintain, and generalize social skills in children with ASD [36]. Furthermore, given the obstacles that the presence of self-injury and cooccurring problem behaviours in autism present to the treatment of the core symptoms of the disorder, it is vital that interventions that primarily target the underlying causes of such problems are identified and implemented in clinical practice. Providing empirically supported behavioral interventions to individuals with autism presenting with self-aggressive behavior, will not only impact directly on the problem behavior, but will increase individuals' social educational, and environmental experiences throughout their lifetime.

## 5. Limitations

The endogenous behaviours were also affected by errors. These are analogous to disturbances, which are sets of unspecified causes of the effect variable. These unobserved variables (E1, E2, and E3) contributed relatively large estimates to the model. Likewise, social skill was also affected by D1, another unspecified variable. It is apparent that these variables contribute to the latent and endogenous variables in the model. These variables could be hereditary or related to other cooccurring disorders or conditions that were not present in this study. Future research could incorporate other potential variables into the model to better account for appropriate and inappropriate social skills. 

## 6. Conclusion

Challenging behavior is more prevalent among individuals with ASD [37] and increases with greater needs for assistance and more restricted receptive and expressive communication [6]. Severe self-injurious behavior can be highly persistent in individuals diagnosed with ASD [6]. Positive social behaviors can lead to positive outcomes for the individual, and social deficits have been shown to impact on the development of further difficulties [14, 22].

If problem behaviors and social deficits such as those examined in this paper cooccur with SIB, then clinicians can develop a greater understanding of the complex relationship of such challenges. Interventions could examine the “coercive trap” that can occur as a result of deficits in one repertoire that negatively affect other repertoires. Furthermore, it has been argued that people with challenging behavior comprise an overmedicated population [38]. Behavioral interventions targeting cooccurring problems and prerequisites to SIB could become more effective and negate the need for pharmacological intervention and the associated side effects.

## Figures and Tables

**Table 1:** Correlation matrix of manifest variables used in the structural equation model.

	(1)	(2)	(3)	(4)	(5)
(1) Frequency of SIB					
(2) Severity of SIB	.565**				
(3) ASD-C-C	.257*	.324**			
(4) MESSY-II hostile	.031	.03	.099		
(5) MESSY-II adaptive/appropriate	−.206*	−.146	−.188	.481**	
(6) MESSY-II inappropriately assertive	.212*	.129	.087	.728**	.421**

^∗^Correlation is significant at the 0.05 level (2-tailed).

^∗∗^Correlation is significant at the 0.01 level (2-tailed).

**Table 2:** Summary of standardized coefficients.

Parameter estimate	Standardized	*P*
Social_skill *←* D1	−5.232	na
Social_skill *←* SIB frequency	−.274	na
Social_skill *←* SIB_severity	−.008	na
Social_skill *←* ASD_CC_sum	−.046	na
Mess_adaptive_appropriate *←* social_skill	1.266	na
Mess_inappropriately_assertive *←* social_skill	−.722	na
Mess_adaptive_appropriate *←* E2	7.296	na
Mess_hostile *←* social_skill	−.381	na
Mess_hostile *←* E1	11.700	na
Mess_inappropriately_assertive *←* E3	5.318	na
SIB_severity *↔* ASD_CC_sum	29.008	.003
E2 *↔* E1	.856	
E2 *↔* E3	1.469	
BPI_total *↔* SIB_severity	19.366	.001
BPI_total *↔* ASD_CC_sum	23.103	.016
E1 *↔* E3	.791	
BPI_total	34.356	.001
SIB_severity	34.162	.001
ASD_CC_sum	234.546	.001

*χ* ^2^ = 3.415, *P* < .05; relative chi square = .854, GFI = .988, CFI = 1.000, RMSEA = .000, and *P* Ratio = .267.

na: not available.

E1: Error 1.

E2: Error 2.

E3: Error 3.
